# The Effect of HER2 Status on Metaplastic Breast Cancer A Propensity Score-Matched Analysis

**DOI:** 10.3389/fendo.2022.874815

**Published:** 2022-06-16

**Authors:** Jin Hu, Yanting Zhang, Fang Dong, Jian Shen, Hengyu Chen, Lei Li, Tao Huang

**Affiliations:** ^1^ Department of Breast and Thyroid Surgery, Union Hospital, Tongji Medical College, Huazhong University of Science and Technology, Wuhan, China; ^2^ Department of Ultrasound, Union Hospital, Tongji Medical College, Huazhong University of Science and Technology, Wuhan, China; ^3^ Department of Pancreatic Surgery, Union Hospital, Tongji Medical College, Huazhong University of Science and Technology, Wuhan, China; ^4^ Department of Breast and Thyroid Surgery, The Second Affiliated Hospital of Hainan Medical University, Haikou, China

**Keywords:** metaplastic breast cancer, post-mastectomy radiotherapy, human epidermal growth factor receptor 2, prognosis, propensity score-matched

## Abstract

**Background:**

The role of human epidermal growth factor receptor 2 (HER2) in metaplastic breast cancer (MBC) patients remains unclear. The present study aimed to evaluate the effect of HER2 status on MBC patients by propensity-score matching (PSM).

**Methods:**

The SEER data from 2010 to 2016 were extracted. The breast cancer-specific survival (BCSS) of MBC patients, diagnosed from 2001 to 2016, was compared using Kaplan–Meier analysis. The multivariate Cox proportional model between groups was performed. PSM was used to make 1:1 case-control matching.

**Results:**

We included 1887 patients with a median follow-up time of 28 months (range 1-83 months). 1749 (92.7%) and 138 (7.3%) patients presented in the HER2-negative group and HER2-positive group. 833 (44.1%) patients received post-mastectomy radiotherapy (PMRT). The HER2-positive group had younger patients, lower tumor grades, and more advanced tumor stages. The prognoses were related to age of diagnosis, race/ethnicity, TNM stage, and PMRT in multivariate Cox analysis. ER status and HER2 status had no impact on BCSS. In the Kaplan-Meier analysis, PMRT was associated with a better prognosis. Importantly, patients with HER2-negative status can benefit from PMRT, but not those with HER2-positive status. After PSM, on multivariate Cox analysis, the prognosis was related to HER2 status and PMRT. In the Kaplan-Meier analysis, PMRT was related to a better prognosis for HER2-negative patients.

**Conclusions:**

Our findings supported that PMRT and HER2-positive status were associated with a better prognosis after PSM. However, HER2-negative, but not HER2-positive patients could benefit from PMRT.

## Background

Metaplastic breast cancer (MBC) was rare and the World Health Organization identified it as a unique pathological type in 2000 (1). MBC is a rare histologic subtype, accounting for about 2-5% of breast cancer (2). It was classified into 5 subtypes: squamous cell carcinoma, spindle cell carcinoma, matrix-producing carcinoma, carcinosarcoma, and metaplastic carcinoma with osteoclastic giant cells (3–7). With the improvement of pathologists’ awareness of MBC, the incidence also increases (8). However, due to its rarity, the role of human epidermal growth factor receptor 2 (HER2) status in the treatments and prognoses of MBC is unclear.

Of note, although treatments of MBC are parallel to that of infiltrating ductal carcinoma (IDC) (9), the prognosis of MBC patients was worse than that of IDC even after receiving comprehensive treatment (10, 11). However, there is no consensus on post-mastectomy radiotherapy (PMRT) in the management of MBC. On the one hand, some researchers reported that PMRT of patients showed a better prognosis than that non-PMRT (9, 12–17). On the other hand, others debated that no connection was presented between PMTR and outcomes (18–21). The management strategy and sample sizes of the study populations may result in this conflict.

HER2-positive (HER2 +) status in traditional breast cancers is an aggressive disease related to drugs resistance, regional recurrence, metastases, and outcomes (22). It had been proved that HER2+ patients that underwent radiotherapy and anti-HER2 therapy had better survival outcomes (23). However, our published report showed that HER2 + patients diagnosed with MBC receiving RT had not a superior breast cancer-specific survival (BCSS) than that not RT (24). This discrepancy may be due to several reasons. Firstly, the characteristics of MBC are different from traditional breast cancer. Secondly, there is no study to explore the role of HER2 status in MBC patients underwent PMRT.

Therefore, to improve the comprehensive treatment of MBC, it is urgent to explore PMRT. Based on the above factors, the information of MBC patients was extracted from the Surveillance, Epidemiology, and End Results (SEER) registry to explore the effect of PMRT on MBC patients under different HER2 statuses.

## Materials and Methods

### Patients

**Figure 1:**
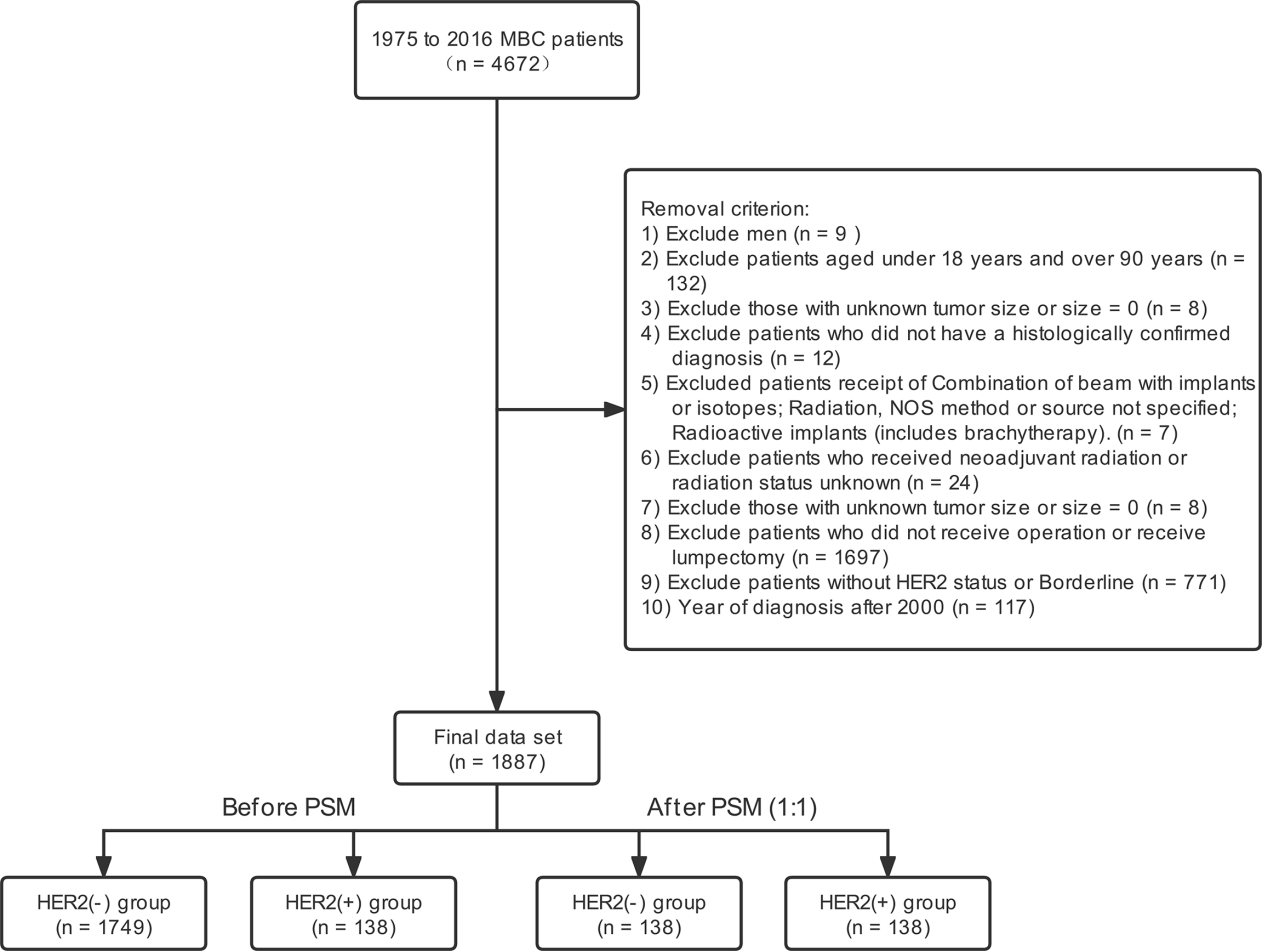
Stepwise inclusion and exclusion counts. MBC, metaplastic breast cancer; PSM, propensity score-matching; Her2, Human epidermal growth factor receptor 2.

Data from 2010 to 2016 were obtained from the SEER database. The demographic and clinicopathological information was obtained from the database. The international classification of diseases for oncology Version 3 (ICD-O-3) codes identified the metaplastic histology, including 8560, 8562, 8570–8572, 8575, and 8980–8982 (24–26). Finally, 1887 patients were included. **Figure 1** showed the inclusion criteria.

### Demographic and Clinicopathologic Variables

Although it is rare, we still include more comprehensive study variables. Demographic variables, including age at diagnosis, race/ethnicity recorded in the SEER database (White, Black, other), and insurance status, were enrolled. The clinical and pathologic variables included grade, histology, tumor size (T1, T2, T3, T4), regional node status (N0, N1, N2, N3), PMRT, post-mastectomy chemotherapy (PMCT), and biomarker parameters (ER, HER2). HER2 status, according to the SEER database, was stratified as HER2-negative and HER2-positive groups.

The BCSS, defined as the date of diagnosis to the date of death from MBC, was considered the primary clinical outcome in our study.

### Detection the Status of ER and HER2

In the SEER database, 1) If ER was reported on multiple tumor specimens, the highest value was recorded; 2) In case sample had any positive, that record is positive; 3) If ER status of all tested invasive specimens was negative, the status of ER was negative whatever ER status was *in situ* specimen; 4) The criterion of ER-positive status was that ≥ 1% cells stained positive; 5) HER2 negative status was defined as staining with a score of 0/1+ by IHC; 6) HER2 positive status was defined as staining with a score of 3+ by IHC; 7) The score of 2+ was interpreted as equivocal. The test of fluorescence *in situ* hybridization or silver *in situ* hybridization order to be performed. Only when the ratio of HER2 to CEP17 was >2.2, the results of HER2 amplification was interpreted as positivity.

### Ethics Statement

Since the patient information in the SEER database has been de-identified, the study was exempted from the approval process of the institutional review committee. In addition, consent papers are not applicable.

### Statistical Analysis

The differences between groups were analyzed by the χ2 test. The univariate Cox proportional hazards model was implemented to evaluate the risk factors of BCSS, and then the variables with P-value < 0.1 and with clinically valuable were included in the multivariate Cox proportional hazards model. The Kaplan–Meier method plotted Survival curves, and the difference between the two group was tested by log-rank. Hazard ratios were showed with 95% confidence intervals (CIs). All statistical analyses were conducted using SPSS (version 24.0; IBM, Armonk, New York, USA). P < 0.05 was statistically significant.

Because of the retrospective design, there was a selection bias when patients were divided into HER2-negative and HER2-positive groups. We compared the clinical and pathologic parameters between the groups and found that those parameters were different, including age of diagnosis, tumor grade, TNM, and PMCT. To reduce the confounding factors and treatment selection bias, propensity score matching (PSM) was conducted (27).

## Result

### Demographic and Clinical Characteristics

The SEER registry recorded 2240 patients diagnosis of MBC from 2010 to 2016. The final sample comprised 1887 cases. In this study, 1749 (92.7%) patients had HER2-negative tumors, and 138 (7.3%) had HER2-positive tumors. The median age of the whole cohort was 63 years (range, 20-89 years). There are more white women (n=1558, 82.6%) and more poorly differentiated patients (n=1341, 71.1%). In addition, 48.6% were stage T2. 1478 (78.3%) and 405 (21.5%) patients had ER-negative and ER-positive status. In terms of treatment, 833 (44.1%) patients underwent PMRT, and 1212 underwent PMCT. Also, 1356 (71.9), 241 (12.8%), 68 (3.6%), and 222 (11.8%) patients diagnosed in N0, N1, N2, and N3 stage. Meanwhile, 427 cases (22.6%) died, including 310 cases (16.4%) related to breast cancer.

The characteristics of clinical and pathological between the two subgroups were showed in **Table 1**. Compared with HER2-negative tumors, HER2-positive tumors were not different concerning race/ethnicity, tumor histology, tumor size, regional node involvement, and PMRT, but HER2-positive patients received more PMCT (P < 0.001). HER2-positive tumors had younger patients (HER2-negative 42.4% vs. HER2-positive 53.6%, P = 0.007) and had higher tumor grade (P < 0.001) than HER2-negative tumors. After PSM, no difference existed between the two groups (**Table 1**).

**Table 1:** Characteristics in MBC patients.

Variables	Before PSM				After PSM		
	HER2(-)	HER2(+)	*p*		HER2(-)	HER2(+)	*p*
Age group			0.007				0.47
≤ 60 years	741 (42.4)	74 (53.6)			67 (48.6)	74 (53.6)	
> 60 years	1008 (57.6)	64 (46.4)			71 (51.4)	64 (46.4)	
Race/ethnicity (n, %)			0.694				0.328
White	1446 (82.7)	111 (80.5)			119 (86.2)	112 (81.2)	
Black	296 (16.9)	26 (18.8)			19 (13.8)	26 (18.8)	
Other	7 (0.4)	1 (0.7)			–	–	
Insurance (n, %)			0.395				0.634
No	281 (16.1)	26 (18.8)			22 (15.9)	26 (18.8)	
Yes	1468 (83.9)	112 (81.2)			116 (84.1)	112 (81.2)	
Grade (n, %)			< 0.001*				0.102*
Well differentiated	81 (4.6)	0 (0)			5 (3.6)	0	
Moderately differentiated	223 (12.8)	8 (5.8)			13 (9.4)	8 (5.8)	
Poorly differentiated	1219 (69.7)	122 (88.4)			112 (81.2)	122 (88.4)	
Undifferentiated	46 (2.6)	2 (1.4)			4 (2.9)	2 (1.4)	
Unknown	180 (10.3)	6 (4.3)					
Histology (n, %)			0.096				0.480*
Metaplastic carcinoma	1534 (87.7)	116 (84.1)			122 (88.4)	116 (84.1)	
Adenosquamous carcinoma	91 (5.2)	14 (10.1)			10 (7.2)	14 (10.1)	
Carcinosarcoma	82 (4.7)	6 (4.3)			6 (4.3)	6 (4.3)	
Others	42 (2.4)	2 (1.4)			0	2 (1.4)	
Tumor size (n, %)			0.240				0.119*
T1	481 (27.5)	41 (29.7)			34 (24.6)	41 (29.7)	
T2	860 (49.2)	57 (41.3)			78 (56.5)	57 (41.3)	
T3	271 (15.5)	25 (18.1)			16 (11.6)	25 (18.1)	
T4	129 (7.4)	13 (9.4)			9 (6.5)	13 (9.4)	
Unknown	8 (0.5)	2 (1.4)			1 (0.7)	2 (1.4)	
Lymph node state			0.083*				0.349
N0	1267 (72.4)	89 (64.5)			91 (65.9)	89 (64.5)	
N1	218 (12.5)	23 (16.7)			24 (17.4)	23 (16.7)	
N2	59 (3.4)	9 (6.5)			3 (2.2)	9 (6.5)	
N3	205 (11.7)	17 (12.3)			20 (14.5)	17 (12.3)	
TNM stage (n, %)			0.022*				0.083*
I	437 (25.0)	37 (26.8)			31 (22.5)	37 (26.8)	
II	1016 (58.1)	64 (46.4)			88 (63.8)	64 (46.4)	
III	230 (13.2)	29 (21.0)			11 (8.0)	29 (21.0)	
IV	50 (2.9)	7 (5.1)			7 (5.1)	7 (5.1)	
Unknown	16 (0.9)	1 (0.7)			1 (0.7)	1 (0.7)	
ER status			0.171				0.087
positive	367 (21.0)	38 (27.5)			26 (18.8)	38 (27.5)	
negative	1382 (79.0)	100 (72.5)			112 (81.2)	26 (18.8)	
PMRT			0.16				0.276
No	983 (56.2)	71 (51.4)			80 (58.0)	71 (51.4)	
Yes	766 (43.8)	67 (48.6)			58 (42.0)	67 (48.6)	
PMCT			< 0.001				0.755
No	649 (37.1)	26 (18.8)			24 (17.4)	26 (18.8)	
Yes	1100 (62.9)	112 (81.2)			114 (82.6)	112 (81.2)	

MBC, metaplastic breast cancer; PSM, propensity score-matching; HER2, Human epidermal growth factor receptor 2; ER, estrogen receptor; PMRT, post-mastectomy radiotherapy; PMCT, post-mastectomy chemotherapy. *Fisher test.

### Prognostic Factors Associated with BCSS

Univariate analysis showed that those parameters were associated with BCSS, including the age of diagnosis, race/ethnicity, insurance, tumor histology, tumor size, and regional node involvement. HER2 status was not related to better BCSS. Interestingly, patients could benefit from PMRT but not PMCT (**Table 2**). After PSM, PMRT also benefits for MBC patients. Tumor size, regional node involvement were associated with a better BCSS.

**Table 2:** Univariate analysis for BCSS in MBC patients.

Variables	Befor PSM				After PSM		
	HRs	95% CI	P		HRs	95% CI	P
Age group							
≤ 60 years	1	[Reference]			1	[Reference]	
> 60 years	1.318	1.048-1.657	0.018		1.142	0.651-2.006	0.643
Race/ethnicity (n, %)							
White	1	[Reference]			1	[Reference]	
Black	1.395	1.063-1.831	0.017		1.072	0.520-2.210	0.850
Other	0.921	0.129-6.567	0.935		–	–	
Insurance (n, %)							
No	1	[Reference]			1	[Reference]	
Yes	0.684	0.496-0.847	0.001		0.483	0.263-0.887	0.019
Grade (n, %)							
Undifferentiated	1	[Reference]			1	[Reference]	
Poorly differentiated	0.774	0.433-1.382	0.387		1.427	0.196-10.361	0.725
Moderately differentiated	0.386	0.190-0.784	0.008		0.674	0.061-7.436	0.748
Well differentiated	0.284	0.107-0.758	0.012		0.876	0.055-14.013	0.925
Unknown	0.692	0.358-1.341	0.276		0.616	0.039-9.851	0.732
Histology (n, %)							
Metaplastic carcinoma	1	[Reference]			1	[Reference]	
Adenosquamous carcinoma	0.211	0.079-0.567	0.002		0.567	0.175-1.832	0.343
Carcinosarcoma	1.637	1.079-2.484	0.021		2.440	0.964-6.177	0.060
Others	0.765	0.341-1.718	0.517		–	–	–
Tumor size (n, %)							
T1	1	[Reference]			1	[Reference]	
T2	2.273	1.519-3.401	<0.001		1.375	0.560-3.372	0.487
T3	7.795	5.177-11.736	<0.001		4.300	1.713-10.790	0.002
T4	13.221	8.591-20.346	<0.001		17.252	6.688-44.503	< 0.001
Unknown	2.219	0.303-16.274	0.433		3.794	0.466-30.863	0.212
Lymph node state							
N0	1	[Reference]			1	[Reference]	
N1	1.846	1.340-2.544	<0.001		2.110	1.023-4.355	0.043
N2	1.993	1.204-3.299	0.007		3.978	1.506-10.507	0.005
N3	1.576	1.090-2.279	0.016		3.288	1.592-6.792	0.001
TNM stage (n, %)							
I	1	[Reference]			1	[Reference]	
II	3.089	1.990-4.795	<0.001		1.326	0.519-3.390	0.555
III	10.396	6.600-16.376	<0.001		6.260	2.447-16.015	<0.001
IV	34.276	20.439-57.479	<0.001		23.360	8.138-67.058	<0.001
Unknown	1.616	0.218-11.969	0.638		4.069	0.489-33.840	0.194
ER status							
negative	1	[Reference]			1	[Reference]	
positive	0.787	0.588-1.053	0.107		0.420	0.179-0.987	0.047
HER2 status							
negative	1	[Reference]			1	[Reference]	
positive	0.784	0.487-1.262	0.316		0.584	0.327-1.044	0.069
PMRT							
No	1	[Reference]			1	[Reference]	
Yes	0.727	0.579-0.913	0.006		0.370	0.196-0.697	0.002
PMCT							
No	1	[Reference]			1	[Reference]	
Yes	0.904	0.718-1.139	0.393		0.754	0.376-1.511	0.426

MBC, metaplastic breast cancer; PSM, propensity score-matching; ER, estrogen receptor; HER2, Human epidermal growth factor receptor 2; PMRT, post-mastectomy radiotherapy; PMCT, post-mastectomy chemotherapy; BCSS, Breast cancer-specific survival; HRs, Hazard ratios; CI, Confidence interval.

the multivariate Cox proportional hazards model was conducted to explore the independent prognostic factors related to BCSS. the results showed that HER2 status was not associated with better BCSS (hazard ratio [HR]: 0.740; 95%CI: 0.453–1.209; P = 0.230), as well as ER status (HR:0.756, 95%CI: 0.562-1.017). Older patients had a worse prognosis (HR: 1.620; 95%CI:1.248–2.102; P < 0.001). In addition, patients could benefit from PMRT (HR: 0.626; 95%CI: 0.489–0.802; P < 0.001) but not PMCT (HR: 0.853; 95%CI: 0.656–1.110; P = 0.237). Independent prognostic factors associated with BCSS including tumor size (T1 as reference; T2, HR:1.132, 95%CI: 0.541-2.369, P =0.743; T3, HR: 3.202, 95%CI: 1.527-6.713; P = 0.002; T3, HR: 2.815, 95%CI: 1.288-6.154; P = 0.010) and regional node involvement (N0 as reference; N1, HR: 1.366, 95%CI: 0.987-1.889; P = 0.060; N2, HR: 1.294, 95%CI:0.811-2.065; P = 0.279; N3, HR: 1.025, 95%CI: 0.718-1.464; P = 0.890). (**Table 3**) After PSM, patients undergoing PMRT (HR: 0.200; 95%CI: 0.089–0.451; P < 0.001) had a better BCSS than patients not undergoing PMRT. Of note, HER2-positive MBC was associated with better prognoses than HER2-negative MBC.

**Table 3:** Multivariate analysis forBCSS in MBC patients.

Variables	Befor PSM				After PSM		
	HRs	95% CI	P		HRs	95% CI	P
Age group							
≤ 60 years	1	[Reference]			1	[Reference]	
> 60 years	1.620	1.248-2.102	<0.001		0.798	0.379-1.677	0.551
Race/ethnicity (n, %)							
White	1	[Reference]			1	[Reference]	
Black	1.440	1.086-1.911	0.011		0.540	0.249-1.174	0.120
Other	0.950	0.116-7.754	0.962		–	–	
Insurance (n, %)							
No	1	[Reference]			1	[Reference]	
Yes	0.825	0.623-1.093	0.180		0.823	0.360-1.878	0.643
Grade (n, %)							
Undifferentiated	1	[Reference]			1	[Reference]	
Poorly differentiated	0.834	0.461-1.509	0.548		1.532	0.170-13.813	0.704
Moderately differentiated	0.630	0.304-1.308	0.215		1.666	0.118-23.598	0.706
Well differentiated	0.936	0.331-2.652	0.901		0.021	0.000-3.194E+14	0.839
Unknown	0.644	0.328-1.266	0.202		0.503	0.026-9.694	0.649
Histology (n, %)							
Metaplastic carcinoma	1	[Reference]			1	[Reference]	
Adenosquamous carcinoma	0.275	0.101-0.754	0.012		0.390	0.091-1.679	0.206
Carcinosarcoma	1.138	0.737-1.757	0.559		1.291	0.399-4.176	0.670
Others	0.989	0.438-2.234	0.978		–	–	
Tumor size (n, %)							
T1	1	[Reference]			1	[Reference]	
T2	1.132	0.541-2.369	0.743		0.715	0.088-5.789	0.754
T3	3.202	1.527-6.713	0.002		3.076	0.356-26.589	0.307
T4	2.815	1.288-6.154	0.010		2.610	0.268-25.421	0.409
Unknown	0.597	0.066-5.400	0.646		0.001	0.000-2.183E+257	0.983
Lymph node state							
N0	1	[Reference]			1	[Reference]	
N1	1.366	0.987-1.889	0.060		1.308	0.559-3.062	0.536
N2	1.294	0.811-2.065	0.279		1.221	0.349-4.276	0.755
N3	1.025	0.718-1.464	0.890		1.931	0.755-4.944	0.170
ER status							
negative	1	[Reference]			1	[Reference]	
positive	0.756	0.562-1.017	0.065		0.283	0.099-0.736	0.051
HER2 status							
negative	1	[Reference]			1	[Reference]	
positive	0.740	0.453-1.209	0.230		0.379	0.192-0.746	0.005
PMRT							
No	1	[Reference]			1	[Reference]	
Yes	0.626	0.489-0.802	<0.001		0.200	0.089-0.451	<0.001
PMCT							
No	1	[Reference]			1	[Reference]	
Yes	0.853	0.656-1.110	0.237		0.414	0.163-1.050	0.063

MBC, metaplastic breast cancer; PSM, propensity score-matching; ER, estrogen receptor; HER2, Human epidermal growth factor receptor 2; PMRT, post-mastectomy radiotherapy; PMCT, post-mastectomy chemotherapy; BCSS, Breast cancer-specific survival; HRs, Hazard ratios; CI, Confidence interval.

### Kaplan-Meier Analysis for Patients Undergoing PMRT

**Figure 2:**
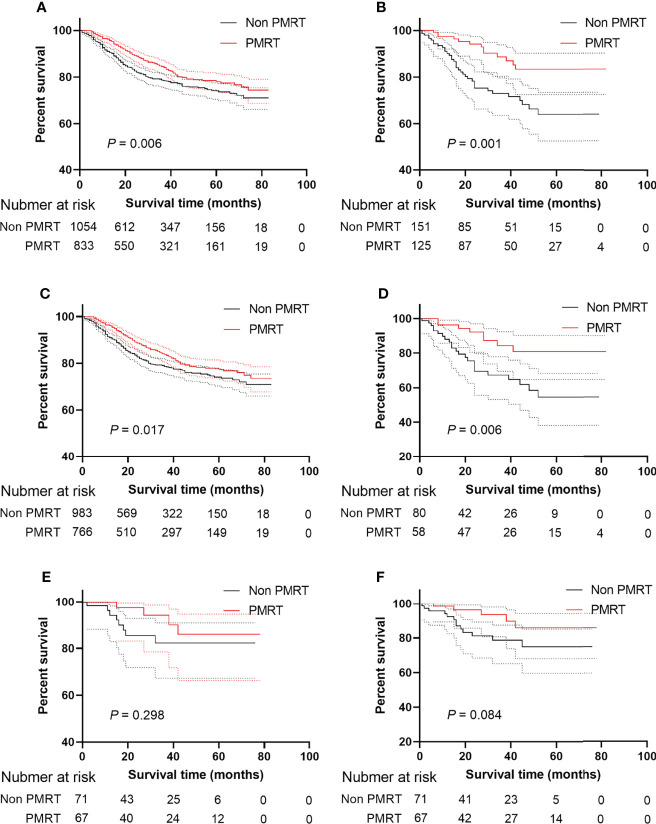
BCSS and OS of MBC patients displayed as Kaplan-Meier curve stratified according to PMRT. **(A)** BCSS curve of Non PMRT group versus PMRT group; **(B)** BCSS curves of Non PMRT group versus PMRT group after PSM; **(C)** BCSS curve of Non PMRT group versus PMRT group patients with Her2 negative status; **(D)** BCSS curves of Non PMRT group versus PMRT group with Her2 negative status after PSM; **(E)** BCSS curve of Non PMRT group versus PMRT group patients with Her2 positive status; **(F)** BCSS curves of Non PMRT group versus PMRT group with Her2 positive status after PSM. MBC, metaplastic breast cancer; BCSS, breast cancer-special survival; PSM, propensity score-matching; PMRT, post mastectomy radiotherapy; Her2, Human epidermal growth factor receptor 2.

The median follow-up time in the HER2 negative group was 28 months (range 1-82 months), and the median follow-up time in the HER2 positive group was 29 months (range 1-78 months). 5-year survival rate in patients receiving PMRT was 78.0% and 74.2% in patients not receiving PMRT (P = 0.001, **Figure 2A**). After PSM, the median follow-up time in the HER2-negative group was 28 months (range, 1–82 months) and the median follow-up time in the HER2-positive group was 29 months (range, 1–78 months). 5-year survival rate was 83.4% in patients receiving PMRT and 64.1% in patients not receiving PMRT (P < 0.001, **Figure 2B**)

### Subgroup Analysis for the Role of HER2 Status in PMRT

To explore the effect of PMRT on MBC patients under different HER2 statuses, this study conducted a subgroup analysis. Patients receiving PMRT had a higher survival rate when HER2 was negative than patients not receiving PMRT in the Kaplan-Meier analysis (P = 0.017, **Figure 2C**) even though after PSM (P = 0.006, **Figure 2D**). When HER2 status was positive, patients receiving PMRT had no better survival than those without PMRT (P = 0.298, **Figure 2E** **)** After PSM, HER2-negative patients could benefit from PMRT. However, HER2-positive patients undergoing PMRT were not associated with better prognoses. (P = 0.084, **Figure 2F**).

## Discussion

Our study explored the role of PMRT in the prognosis of MBC patients and verified the effectivity of HER2 status in prognosis. After propensity score matching, our results showed that PMRT and HER2-positive status were associated with a better prognosis. However, only HER2-negative patients could benefit from PMRT.

The effectiveness of radiotherapy (RT) on MBC is still controversial. Jung et al. (28) reported that RT was not associated with a better prognosis. Those patients’ information was extracted from the Center for Breast Cancer Database and they diagnosed from 2001 to 2008. However, only 35 patients were diagnosed with MBC in those studies. Cecilia et al. (15) included stage I–III MBC patients diagnosed from 2010 to 2014. They illustrated that RT was associated with improved survival. The reasons for this effect could be the fact that, firstly, the sample size of the study varies greatly. Secondly, different eras might exist different results. As pathologists’ understanding and surgeons’ recognition of MBC has been improved, the prognosis might have also been improved.

As we all know, to minimize local recurrence after patients undergoing lumpectomy, post-surgery radiotherapy is considered as a standard component of lumpectomy to treat patients with IDC. Dave et al. (29) and Yu et al. (10) found that patients receiving lumpectomy but not total mastectomy can benefit from radiotherapy. The National Comprehensive Cancer Network breast cancer guidelines recommended that the T1-2N1 stage patients should receive PMRT, while those with stage N2 might undergo PMRT (30). In addition, 5-year survival rates of the MBC patients ranged from 49 to 83%, which suggested that the effect of PMRT in those tumors is not clear. In the present study, PMRT was associated with a better prognosis for MBC patients.

According to previously published studies, the rate of MBC underwent CT ranged from 33 to 86% (31–33). The reasons might be that, firstly, the widely gapped rates might suggested that the effectivity of patients underwent CT remained unclear, but some studies with small sample size showed that patients receiving CT had a superior prognosis (34–36). Secondly, the high rate may be that the triple-negative phenotype was the common molecular subtype of MBC, which is characterized by more aggressive cancer (37). The next but not the last reason is that in the NCCN guideline, its treatment was paralleled to that of IDC (38). Nevertheless, CT can not affect the prognosis of MBC patients, which is supported by most researchers (28, 39–41). 64.2% of patients received CT but they had no better outcomes than that not receiving CT, in the present study, which was consistent with the previous study (15, 42). The presence of more than one metaplastic component may be one of reasons for chemotherapy-resistant.

Although the triple-negative phenotype was the common molecular subtype in MBC, HR-positive and HER2 over-expression tumors do exist (43).

A published study reported that HER2 status was associated with a better prognosis for MBC patients (37). This is in contrast to invasive ductal and lobular carcinoma of the breast (22). Interestingly, some small sample reports suggest that anti-estrogen therapy does not improve the disease-free and overall survival of HR-positive MBC (8, 32, 44). In our study, HER2-positive status was associated with better outcomes, this conclusion is consistent with a recent study by Schroeder et al. that was published. Additionally, little is known about the presentations and prognoses of HER2 positive MBC, due to lack of reports of tumor HER2 receptor status. There was a particularly significant gap when consider the availability and use of HER2-directed therapy. In addition, by investigating the response of MBC to HER2 targeted therapy, we can understand the vulnerability to antibodies (37).

Owing to the rarity of HER2 over-expression tumors, clinicopathologic features need to be fully determined. The incidence of MBC is unknown, so the association of these therapeutic factors with MBC is unknown. Her2-positive breast cancer is an invasive disease, and until recently the overall survival rate for this subtype of breast cancer had been the worst (45, 46). Overall survival in this subtype had been greatly improved due to the use of HER2-targeted therapies by antibody-based approaches (e.g., trastuzumab, pertuzumab) and small-molecule inhibitors (lapatinib, neratinib) (47, 48). However, the involvement of HER2 over-expression in MBC prognoses is unknown. Previous studies have found the rate of HER2 over-expression ranging from 0% to 25% (49, 50). In our study, 7.3% of MBC patients had HER2 over-expression, which is consistent with previous studies. According to the current consensus guidelines, the degree of HER2 overexpression or amplification was thought to be intermediate between typical breast cancer and MBC, as reported in previous studies (51).

Our study has several key strengths. The role of HER2 status and PMRT in the prognosis of MBC is unclear. From our results, the prognosis was improved in MBC patients receiving PMRT. In addition, HER2 status can redefine the role of PMRT in the prognosis of MBC.

Our study has several limitations. First, due to its retrospective study, it is characterized by the nature of observation and the possibility of selection bias. Second, the SEER database lacks information on hormone therapy, anti-Her-2 therapy, and baseline characteristics including working status, comorbidity, and socio-economic environmental parameters. Third, the SEER database can not provide detailed chemotherapy and radiotherapy information, so it is impossible to conduct further case-control studies. However, our results will help researchers understand the role of HER2 in the prognosis of MBC.

## Conclusions

Our findings supported that, after PSM, PMRT and HER2-positive status were associated with a better prognosis. However, only HER2-negative patients could benefit from PMRT.

## Data Availability Statement

The datasets presented in this study can be found online at https://doi.org/10.6084/m9.figshare.19800526.

## Author Contributions

We sincerely appreciate our department members for providing great support. Thanks are due to TH, LL, and HC for their conception and design of this study and to YZ for her help with the methodology. During the period of writing and revising our manuscript, FD and JS had given us many good suggestions, thanks sincerely. All authors contributed to the article and approved the submitted version.

## Funding

This study was supported from the National Science Foundation Committee (NSFC) of China (Grant number: No. 81702397 to LL). This project is supported by Hainan Province Clinical Medical Center.

## Conflict of Interest

The authors declare that the research was conducted in the absence of any commercial or financial relationships that could be construed as a potential conflict of interest.

## Publisher’s Note

All claims expressed in this article are solely those of the authors and do not necessarily represent those of their affiliated organizations, or those of the publisher, the editors and the reviewers. Any product that may be evaluated in this article, or claim that may be made by its manufacturer, is not guaranteed or endorsed by the publisher.

## Abbreviations

MBC, Metaplastic breast cancer; HER2, Human epidermal growth factor receptor 2; IDC, Invasive ductal carcinoma; PMRT, Post-mastectomy radiotherapy; SEER, Surveillance, Epidemiology, and End Results; ICD-0-3, International Classification of Diseases for Oncology Version 3; PMCT, Post-mastectomy chemotherapy; BCSS, Breast cancer-specific survival; ER, Estrogen receptor; HRs, Hazard ratios; CI, Confidence interval.
